# An Inside Job: Molecular Determinants for Postsynaptic Localization of Nicotinic Acetylcholine Receptors

**DOI:** 10.3390/molecules26113065

**Published:** 2021-05-21

**Authors:** Michael Ferns

**Affiliations:** Departments of Anesthesiology & Pain Medicine, and Physiology & Membrane Biology, University of California Davis, Davis, CA 95616, USA; mjferns@ucdavis.edu

**Keywords:** neuromuscular junction, autonomic synapse, cholinergic synapse, receptor localization, structural motif, binding partner, scaffolding protein

## Abstract

Nicotinic acetylcholine receptors (nAChRs) mediate fast synaptic transmission at neuromuscular and autonomic ganglionic synapses in the peripheral nervous system. The postsynaptic localization of muscle ((α1)_2_β1γδ) and neuronal ((α3β4)_2_β4) nicotinic receptors at these synapses is mediated by interactions between the nAChR intracellular domains and cytoplasmic scaffolding proteins. Recent high resolution structures and functional studies provide new insights into the molecular determinants that mediate these interactions. Surprisingly, they reveal that the muscle nAChR binds 1–3 rapsyn scaffolding molecules, which dimerize and thereby form an interconnected lattice between receptors. Moreover, rapsyn binds two distinct sites on the nAChR subunit cytoplasmic loops; the MA-helix on one or more subunits and a motif specific to the β subunit. Binding at the latter site is regulated by agrin-induced phosphorylation of βY390, and increases the stoichiometry of rapsyn/AChR complexes. Similarly, the neuronal nAChR may be localized at ganglionic synapses by phosphorylation-dependent interactions with 14-3-3 adaptor proteins which bind specific motifs in each of the α3 subunit cytoplasmic loops. Thus, postsynaptic localization of nAChRs is mediated by regulated interactions with multiple scaffolding molecules, and the stoichiometry of these complexes likely helps regulate the number, density, and stability of receptors at the synapse.

## 1. Introduction

Nicotinic acetylcholine receptors (nAChRs) are ligand-gated ion channels belonging to the cys-loop superfamily that includes GABA_A_, glycine, and 5HT_3_ serotonin receptors. In the peripheral nervous system (PNS), nAChRs mediate fast synaptic transmission at neuromuscular and autonomic ganglionic synapses, where they are localized in the postsynaptic membrane [1]. In the CNS, nAChRs mediate diverse and mainly neuromodulatory functions and are usually localized in presynaptic terminals where they modulate the release of neurotransmitters such as glutamate, GABA, and norepinephrine [2,3]. Thus, nAChRs play important roles in the functioning of both the peripheral and central nervous systems [3,4]. Consistent with this, dysfunction of nAChRs is a contributing factor or the direct cause of a variety of human neurological disorders. In the neuromuscular system, autoimmune antibodies to the nAChR lead to myasthenia gravis [5], and genetic mutations in the nAChR cause congenital myasthenic syndrome (CMS) [6], both of which are characterized by impaired synaptic transmission and muscle weakness. In the CNS, mutations in the neuronal nAChR cause autosomal dominant nocturnal frontal lobe epilepsy, and declines in nAChR expression contribute to Alzheimer’s disease, Parkinson’s disease, and schizophrenia (reviewed in [3,4,7]). Moreover, nAChRs mediate the effects of nicotine and upregulation of receptors occurs as part of tobacco addiction.

Nicotinic AChRs are pentameric channels that occur in multiple subtypes, formed from different combinations of subunits that convey different pharmacological and physiological properties [2,7]. The subunits are subdivided into two groups: Alpha subunits (α1–10) which contain conserved cysteine residues in the extracellular domain and function in ligand binding, and non-alpha subunits (β1–4, γ, δ, and ε) which function as accessory subunits. Muscle nAChR at the neuromuscular junction (NMJ) is composed of 2 α, and one β, δ, and γ (fetal) or ε (adult) subunits. Neuronal nAChR are either homo-pentamers composed of α7, α8 or α9 subunits or hetero-pentamers composed of α2–α6 and β2–β4 subunits. Different combinations of alpha and beta subunits generate neuronal nAChRs with diverse stoichiometries although all receptors contain 2–3 alpha subunits. These differences in channel subunit composition are thought to play important roles in modulating the gating and biophysical properties of the channels, as well as the protein interactions involved in their trafficking and localization.

Alpha and non-alpha subunits share considerable homology and all subunits have a similar structure consisting of a large extracellular N-terminal domain, four transmembrane domains that form the central channel pore, and a large cytoplasmic loop between transmembrane domains 3 and 4. The structure and function of the extracellular and pore domains of nicotinic acetylcholine receptors are discussed in detail in other articles in this issue. The focus of this article is to review the structure of the intracellular domain and its role in mediating the postsynaptic localization of nicotinic receptors at fast cholinergic synapses in the PNS [4,8]. The main emphasis is on muscle nAChRs, for which the molecular basis for localization is best understood. However, neuronal nAChRs are also briefly reviewed in order to point out some intriguing parallels, and differences, in their mode of postsynaptic localization.

## 2. Structure of the nAChR Intracellular Domain

**Figure 1:**
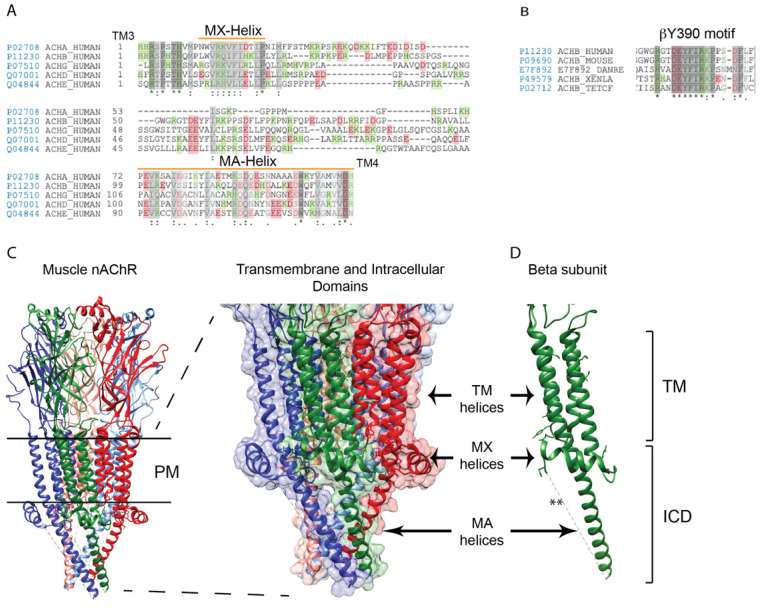
Structure of the muscle nAChR intracellular domains. (**A**) A sequence alignment of the muscle nAChR subunit large cytoplasmic loops between transmembrane domains 3 and 4. The greatest similarity between subunits occurs in the proximal and distal portions of the loop, which contain the MX- and MA-α-helices, respectively (green and red colors denote positively and negatively charged amino acids, respectively. Identical residues are denoted by * and conserved and semi-conserved substitutions by : and . respectively. (**B**) The mid-portion of some subunit loops contain motifs that are highly conserved across species, such as the sequence surrounding β subunit tyrosine 390 (Y390). (**C**) A high resolution structure of the Torpedo (muscle-type) nAChR (PDB: 6UWZ), with an enlarged view of the transmembrane and intracellular domains is shown to the right (red, α subunit; green, β; blue, δ). All subunits contain an MX-helix that is positioned laterally near the plasma membrane, and an MA-helix that extends ~40 Å down from the membrane. The mid-portion of each subunit loop is not resolved and presumed to be disordered. (**D**) A corresponding view of the β subunit alone, shows the MX and MA-helices and the missing intervening structure (**) (abbreviations: PM: Plasma membrane; TM: Transmembrane; ICD: Intracellular domain).

Nicotinic receptor subunits contain two cytoplasmic loops; a short loop (of ~8 amino-acids) between TM1 and TM2 and a large loop of between 110 and 150 amino-acids between TM3 and TM4. The TM1–2 loop is relatively conserved, whereas the TM3–4 loop is the most divergent region between different receptor subunits (for a review, see [9]). For the TM3–4 loop, the greatest similarity between subunits lies in the proximal part of the loop following TM3 and the distal part of the loop prior to TM4, which both contain predicted alpha-helical secondary structures (Figure 1A). The intervening, mid-section of the loop is more variable in length and sequence in different subunits and meaningful alignments cannot be made for this region. Notably, however, some receptor subunits contain stretches of amino acids in the central loop region that are highly conserved across species (Figure 1B), suggesting that important subunit-specific functions may be mediated by these motifs [9].

High resolution structures have now been obtained for several members of the cys-loop family including the muscle nAChR (Figure 1C) [10,11,12] neuronal α4β2, α3β4, and α7 nAChRs [13,14,15], GABA_A_ receptor [16], glycine α3 receptor [17], and 5HT_3_ receptor [18]. These reveal a conserved architecture of the channel and constituent subunits [19,20], as well as providing new insights into the structural basis of transmitter binding and channel gating. One region that is absent or only partially resolved in these structures, however, is the large cytoplasmic loop domain of each subunit (Figure 1C). Although structural information is limited, two separate alpha helices have been identified in the large cytoplasmic loop of some cys-loop receptors. The MX-helix is apparent in high resolution structures of the 5HT_3_ receptor and muscle and neuronal nAChRs, and is present in all subunits of those pentamers [11,15,16,19]. This short helix (~13 amino acids) lies just after TM3 and is positioned laterally in the proximal TM3–4 cytoplasmic loop (Figure 1C), placing it in close proximity to the inner leaflet of the plasma membrane. Interestingly, it ends near the interface with the adjacent receptor subunit and may contribute to the cytoplasmic interface between subunits. The MA-helix was first resolved in structures of the muscle nAChR derived from cryo-electron microscopy [12], and later confirmed in high resolution structures of the 5HT_3_ receptor and neuronal nAChRs [11,15,16,19]. This long, amphipathic helix lies in the distal portion of the cytoplasmic loop and is continuous with the TM4 helix (Figure 1C,D). The MA helices of each subunit extend approximately 40 Å down from the plasma membrane and frame a conical intracellular vestibule that lies beneath the channel pore. Interestingly, this vestibule contains lateral portals for ion efflux near TM4 and charged residues in this region contribute to the ion selectivity of the channel [10,21].

The structure of the intervening sequence between the MX and MA helices is not resolved for any cys-loop receptor subunits and is likely disordered, as suggested from secondary structure predictions. Several highly conserved phosphorylation sites and antibody binding sites map to this mid-loop region in some muscle and neuronal nAChR subunits, suggesting it is partially solvent-exposed, and able to interact with kinases and possibly other cytoplasmic proteins.

## 3. Postsynaptic Localization of the Muscle nAChR

The muscle nAChR mediates fast synaptic transmission at the NMJ on skeletal muscle fibers. Transmission at this synapse is rapid and reliable, with a large safety factor under normal physiological conditions. This stems from the immense size of the NMJ, where an action potential triggers the release of many synaptic vesicles (~25–50) at multiple active zones in the presynaptic motor nerve terminal. In addition, the muscle nAChR is localized at an extremely high density (~10,000 per μm^2^) at the top of the postsynaptic junctional folds. Consequently, the released acetylcholine directly gates thousands of postsynaptic nAChRs, evoking a large depolarization (endplate potential) that exceeds the threshold and triggers an action potential in the muscle fiber. The number and density of nAChRs is a key determinant of synaptic efficacy, therefore, and defects in nAChR expression in genetic and autoimmune diseases impair transmission and result in myasthenia [22,23]. Not surprisingly, therefore, the expression levels of nAChR at the NMJ are tightly regulated and reflect multiple mechanisms including clustering, anchoring, and stabilization of receptors in the postsynaptic membrane [8]. These processes are controlled by specific protein interactions between molecular determinants in the acetylcholine receptor and postsynaptic scaffolding and signaling proteins. In the vertebrate nAChR, the molecular determinants that regulate these inter-related aspects of receptor localization lie within the major cytoplasmic TM3–4 loop.

The specific, high density localization of nAChRs in the postsynaptic membrane is established during formation of the NMJ and is regulated by a nerve-derived signal called agrin. During synapse formation, motoneuron-derived agrin signals via a receptor complex on muscle cells consisting of the MuSK receptor tyrosine kinase [24] and LRP4 co-receptor [25,26] to induce and/or maintain nAChR clusters at nerve-muscle contacts. This occurs via a complex signaling pathway that includes Dok7 [27,28], Tid1 [29], crk [30], pak [31], and the small GTPases, Rac, cdc42, and Rho [32,33]. Indeed, mice with targeted deletions of agrin, MuSK, LRP4, and Dok7 all fail to form functional NMJs and die shortly after birth [28,34,35,36]. Intriguingly, the nerve-evoked activity has an antagonistic role to agrin during synapse formation, and disperses prepatterned nAChR aggregates that are not contacted by nerve terminals [37,38,39,40,41].

### 3.1. AChR Localization Is Mediated by Rapsyn

The key downstream component of the agrin signaling pathway that mediates nAChR localization is the peripheral membrane protein rapsyn. Rapsyn colocalizes precisely with the AChR in Torpedo electric organ and at developing and adult vertebrate NMJs [42,43,44]. Moreover, biochemical studies on purified synaptic membranes from the Torpedo electric organ estimate that the stoichiometry of rapsyn to AChR is in the range of 0.5–2 [45,46]. When expressed in heterologous cells, rapsyn self-aggregates and co-clusters the nAChR as well as several other postsynaptic proteins on the cell surface [47,48]. Conversely, in rapsyn-deficient mice, the nAChR and several other proteins fail to cluster at the NMJ, resulting in non-functional synapses and perinatal lethality [49]. Multiple mutations in the RAPSN gene have also been identified in humans that cause congenital myasthenic syndrome. These mutations typically result in decreased nAChR levels at the synapse and thereby impair neuromuscular transmission and muscle function [50,51]. Thus, rapsyn is sufficient for nAChR clustering in vitro and necessary for nAChR localization at the NMJ in vivo.

Rapsyn is a 43 kD protein with a predicted domain structure that includes a myristylation signal at the N-terminus that targets it to the membrane, seven tetratricopeptide repeats, a coiled-coil domain, a RING-H2 domain, and a C-terminus containing highly conserved serine phosphorylation sites. The functional analysis using rapsyn deletion constructs expressed in heterologous cells suggest that the seven tetratricopeptide repeats mediate rapsyn self-association [52,53], the coiled-coil domain interacts with the nAChR [52], and the cysteine-rich RING structure interacts with scaffold proteins and also has E3 ligase activity [54]. However, this functional analysis of rapsyn’s domains is inconsistent with the defects observed in several rapsyn CMS mutants (reviewed in [55,56]), and the rapsyn domains that mediate many of its key interactions, including binding the nAChR, await further confirmation. In addition, rapsyn binds the MuSK- [57] and dystroglycan-based transmembrane scaffolds [58,59], as well as multiple cytoskeletal-associated proteins including alpha-actinin [60], MACF1 [61,62], and plectin-1f [63]. Taken together, these findings suggested a model in which rapsyn binds the nAChR with 1:1 stoichiometry and recruits it to a stable postsynaptic scaffold established by rapsyn dimerization and rapsyn links to other postsynaptic scaffolding and cytoskeletal proteins. While this simple model has prevailed for over 25 years, recent studies suggest that nAChR localization occurs through considerably more complex and regulated rapsyn interactions.

### 3.2. AChR-Rapsyn Stoichiometry

A large body of evidence has now accumulated and converged to demonstrate that rapsyn interacts with the nAChR in a regulated and variable stoichiometry of ≥2:1, rather than an invariant 1:1 interaction. First, nAChR initially associates with rapsyn in the late secretory pathway and they are co-transported as a pre-formed complex to the cell surface [64,65,66]. Indeed, rapsyn transport to the plasma membrane (PM) is dependent on the nAChR and rapsyn is retained in the Golgi apparatus in myoblasts and zebrafish mutants that lack the receptor [67,68]. Pre-formed rapsyn/nAChR complexes do not automatically aggregate on the cell surface, however, and remain diffusely distributed in denervated muscle or cultured myotubes [69]. Thus, nAChR clustering requires either additional signals that induce rapsyn dimerization [70], and/or binding of additional rapsyn molecules to the receptor. Second, it was found that the ratio of rapsyn to nAChR increases during maturation of the NMJ [71,72], and that overexpression of rapsyn in muscle stabilizes and increases the packing density of the nAChR [73]. This implies that additional rapsyn molecules can be recruited to free sites on the nAChR, although rapsyn binding to other proteins cannot be discounted. Third, rapsyn has been shown to interact with multiple receptor subunits in heterologous cell expression experiments [74,75,76], suggesting that several rapsyn binding sites are available on the receptor. Fourth, several groups found that agrin-MuSK signaling increases the amount of rapsyn associated with surface nAChR by around 1.7-fold [69,77,78,79]. As the amount of nAChR co-immunoprecipitated with rapsyn does not increase, this argues for an increase in the number of rapsyn molecules bound to each receptor, rather than an increase in their binding affinity. Fifth, a cryo-electron tomography study of Torpedo postsynaptic membranes detected 1–3 rapsyn molecules per receptor, which form an interconnected lattice between nAChRs [80]. Taken together, these studies provide compelling evidence that rapsyn binds the nAChR with a regulated and variable stoichiometry of ≥2:1 to mediate its postsynaptic localization.

### 3.3. The nAChR-Rapsyn Binding Sites

Attempts to identify the specific determinants in the nAChR subunit cytoplasmic loops that bind rapsyn have proven extremely difficult for a number of reasons. Multiple binding sites create redundancy in rapsyn/nAChR association, and deletions or mutations within the cytoplasmic loops often impair receptor assembly and surface expression. These issues mask or prevent the identification of rapsyn binding sites on intact nAChR. Biochemical approaches using isolated loop fragments have also been hampered by rapsyn’s notorious insolubility and tendency to self-aggregate. To circumvent these issues our group utilized chimeric proteins consisting of the CD4 single-pass transmembrane protein fused to the large cytoplasmic loop of each nAChR subunit to map rapsyn’s binding sites. This approach has been used in multiple studies to express isolated cytoplasmic domains of ion channels at the plasma membrane, and to map their targeting and localization signals and protein interactions [81,82]. When expressed in heterologous cells, we found that rapsyn clustered and cyto-skeletally anchored CD4-α, β, and ε subunit loops, although with different efficiencies [76]. Rapsyn interacted most strongly with the β subunit, which is consistent with previous cross-linking [46] and yeast two-hybrid studies [58] that also identified an interaction between rapsyn and the β subunit. Using β loop fragments, the rapsyn binding site was mapped to the MA α-helix in the C-terminal portion of the loop, and this interaction was independent of agrin signaling in muscle cells [76]. The MA α-helical structure is present in all muscle nAChR subunits, although there is only moderate similarity in the primary sequence of the α-helix in different subunits (Figure 1A). Together with the observation that rapsyn interacts more strongly with the complete MA-helix compared to its first or second halves, this suggests that rapsyn likely recognizes structural features of the helix rather than a specific amino acid sequence. This would also explain why rapsyn can interact with multiple muscle nAChR subunits, and even with some other members of the cys-loop receptor family such as α4β2 and α7 nAChRs [83,84] and GABA_A_ receptors [85], which possess analogous MA helices. These findings identify up to four potential rapsyn–MA-helix binding sites on the AChR, but it remains unclear how many are actually utilized and if they are required for AChR localization. Unfortunately, it is not possible to delete or mutate the MA-helix of multiple subunits as this prevents receptor assembly. However, a three codon deletion in the β loop α-helix has been linked to a congenital myasthenic syndrome with severe nAChR deficiency that is consistent with both the defective interaction with rapsyn and impaired assembly [86]. Moreover, overexpression of a soluble MA-helix fragment in cultured muscle cells prevents agrin-induced clustering of the nAChR [87]. Thus, it seems likely that rapsyn binding to the MA helix of one or more subunits plays a critical role in postsynaptic aggregation of the nAChR.

In addition to the MA-helix, a distinct and regulated rapsyn binding site has also been identified on the nAChR β subunit. Remarkably, when CD4-subunit loop chimeras are individually expressed in cultured muscle cells, only CD4-β loop is recruited to agrin-induced AChR clusters [77]. Progressive deletions within the β loop identified a 20 amino-acid sequence that was sufficient for postsynaptic localization and this sequence contains a highly conserved tyrosine residue whose phosphorylation is induced by agrin signaling [88]. Indeed, agrin-induced phosphorylation of βY390 induced rapsyn binding to the β loop motif, and conversely mutation of Y390 abolished both clustering of the CD4-β loop fragment and its interaction with rapsyn. In analogous experiments on intact AChR in muscle cells, agrin increased the ratio of rapsyn binding to wild type nAChR but not to nAChR with a mutated Y390 phosphorylation site (AChR-β^3F/3F^) [77]. Together, these findings identify a 20 amino-acid motif in the first half of the β subunit cytoplasmic loop which forms a distinct, phosphorylation-dependent binding site for rapsyn. As might be expected given that rapsyn also binds the nAChR subunit MA-helix, rapsyn binding to the βY390 motif is not essential for nAChR localization at the NMJ. Notably, however, AChR-β^3F/3F^ knock-in mice have simplified and smaller NMJs with a significantly reduced density and number of postsynaptic nAChRs [89]. Thus, regulated binding of rapsyn to this distinct site on the proximal β subunit cytoplasmic loop clearly contributes to nAChR localization.

**Figure 2:**
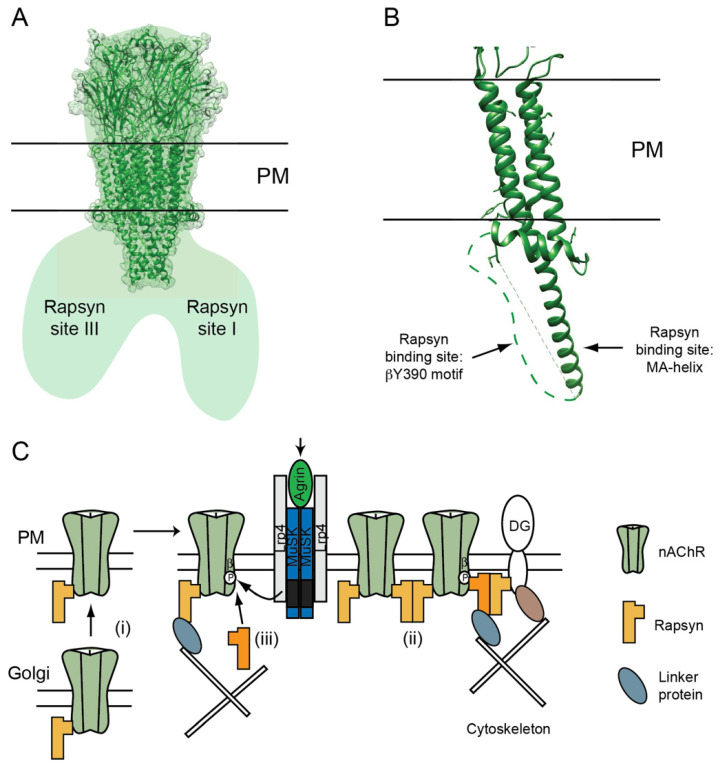
nAChR–rapsyn binding sites. (**A**) Structural studies on Torpedo synaptic membranes showed that each nAChR is associated with 1-3 rapsyn molecules, which bind at two homologous sites on adjacent subunits (site I and II), and a non-homologous site on the opposing face of the receptor (site III) [80]. (**B**) Complementary studies using subunit loop chimeric proteins identified rapsyn binding sites on the MA-helices and a phosphorylation-dependent binding site on the conserved βY390 motif. (**C**) These findings suggest a revised model where rapsyn localizes the nAChR in the postsynaptic membrane via multiple, regulated interactions. Rapsyn associates with the nAChR in the Golgi apparatus (i) and they exist as pre-formed complexes on the PM. Agrin-MuSK signaling induces their co-clustering via rapsyn dimerization (ii) and by binding of an additional rapsyn molecule to the phosphorylated βY390 motif (iii). Recruitment of additional rapsyn may require a chaperone protein (not shown). These two mechanisms create rapsyn bridges between nAChRs and also anchor receptors to transmembrane and cytoskeletal scaffolding proteins. Thus, the stoichiometry of rapsyn/nAChR complexes is an important determinant of the density and stability of nAChRs in the postsynaptic membrane.

Additional, compelling evidence for multiple rapsyn binding sites on the nAChR comes from structural studies of Torpedo electric organ, a model system for the NMJ. In a 4.6 Å structure of nAChR/rapsyn complexes derived from electron microscopy images of tubular Torpedo membranes, rapsyn showed a 2-fold symmetry, implying two rapsyn molecules associated with a single nAChR [11,90]. Similarly, cryo-electron tomography and subtomogram averaging of Torpedo postsynaptic membranes showed that nAChRs are connected by up to three rapsyn bridges [80]. The rapsyn molecules bind at three stereotyped positions on the nAChR, although the resolution (~4 nm) was not sufficient to identify the receptor subunits involved (Figure 2A). Sites I and II appear to be homologous and likely occur on adjacent subunits, as they are separated by 72° in the pentamer. Site III appears distinct as it is located closer to the membrane and is separated from site I by 108°. The occupancy of each site varied between nAChRs, with binding at site II being the most common. The rapsyn lobes associated with each nAChR also occurred in two classes, whose size is consistent with a rapsyn monomer and dimer, respectively. In most cases, however, rapsyn dimers were observed connecting adjacent receptors and thereby creating an inter-connected lattice of nAChRs in the postsynaptic membrane. These findings suggest that up to three rapsyn binding sites are utilized on the nAChR, which fall in two classes [80]. The simplest possibility is that the homologous sites (I and II) correspond to the MA helix on adjacent subunits, and that the non-homologous site (III) corresponds to the βY390 motif (Figure 2B). However, this raises the question of why rapsyn binds only two of the four potential MA helix sites that were identified on the alpha, beta, and epsilon subunits. One possible explanation is that rapsyn binds to the MA helices at the interface between subunits and this confers additional specificity to the interaction that restricts binding to two sites on the nAChR.

Although the specific subunits that interact with rapsyn remain to be defined, these findings further support a more complex model for nAChR localization where rapsyn–nAChR association occurs via two distinct sites and with a variable stoichiometry of ≥2:1 (Figure 2C). It also raises the possibility that rapsyn binding to each site has differing functions, which combine to localize the receptor in the postsynaptic membrane.

### 3.4. Function of nAChR-Rapsyn Interactions

Rapsyn mediates nAChR localization at developing NMJs through multiple mechanisms, including clustering, anchoring, and metabolic stabilization of the nAChR. These inter-related functions are regulated by agrin-MuSK signaling, and likely relate to the site and stoichiometry of rapsyn binding to the receptor. First, rapsyn’s clustering of nAChR in the PM is thought to be due to its ability to dimerize and thereby cross-link pre-formed rapsyn/nAChR complexes (Figure 2C). Higher ratios of rapsyn molecules per AChR would increase the number of cross-links between nAChRs, leading to an increase in the packing density. Indeed, increased receptor density has been observed with rapsyn overexpression in mouse muscle [71,73], and decreased density is observed in AChR-β^3F/3F^ knock-in mice that lack the phosphorylation-dependent βY390 binding site for rapsyn [89]. Similarly, nAChR packing density in purified Torpedo synaptic membranes also correlates with rapsyn abundance [80]. Second, rapsyn anchors the nAChR to several components of the postsynaptic cytoskeleton and also to transmembrane scaffolding proteins [35,57,59,60,61,62,91]. Again, this is likely enhanced by higher stoichiometries of rapsyn binding, which could allow for more links to the postsynaptic scaffold (Figure 2C). Rapsyn binding to the βY390 motif may be especially important, as mutation of this site increases the detergent extractability of the receptor, implying that it is anchored less effectively to the postsynaptic cytoskeleton [88,89]. Third, rapsyn increases the metabolic stability of nAChR in cultured heterologous and muscle cells [92,93] and at the NMJ [71,73]. In heterologous cells this appears to be mediated by rapsyn binding to the MA-helix, as it does not require beta subunit tyrosine phosphorylation [92]. In muscle cells, agrin-induced stabilization of the nAChR requires βY390 phosphorylation but not clustering, suggesting that it involves rapsyn binding specifically to the Y390 motif [93]. Presumably both rapsyn interactions would combine to stabilize the nAChR and decrease its turnover at the NMJ (Figure 2C). Interestingly, rapsyn’s own turnover is considerably more rapid (~3 d) than that of the nAChR (~11–14 d), placing it in a unique position to regulate receptor turnover [94,95]. Consistent with this, rapsyn levels and stability are regulated by multiple mechanisms including stabilization by binding to HSP90β [79] and CUL3/KLHL8-dependent ubiquitination and degradation by the proteasome [96].

The overall conclusion from these studies is that postsynaptic clustering, anchoring, and stabilization of the AChR are regulated in part by the stoichiometry of rapsyn/AChR complexes. While some functions may stem from a simple increase in the number of rapsyn molecules bound per nAChR, other functions might require rapsyn binding to a specific site (MA helix or βY390 motif). In the latter case, conformational differences between rapsyn bound at each site could influence its possible protein binding partners and thereby its functional effects.

Adding further complexity to the rapsyn/nAChR story, recent findings suggest that rapsyn may also regulate nAChR clustering by an entirely novel mechanism. Rapsyn was found to have E3 ligase activity that is mediated by its C-terminal RING domain, and mutation of this domain abolishes rapsyn-mediated nAChR clustering in both heterologous and muscle cells [54]. Interestingly, rapsyn’s E3 ligase activity mediates neddylation (a modification similar to ubiquitination) of the nAChR δ subunit, and genetic and pharmacological manipulations that block neddylation, as well mutation of the main neddylation site in the δ subunit cytoplasmic loop all impaired nAChR clustering. These results are intriguing although the precise mechanism by which neddylation contributes to nAChR clustering remains unclear [70]. The simplest possibility is that the neddylated δ subunit forms a binding site for rapsyn or another scaffolding protein involved in receptor localization. This seems unlikely, however, as no evidence has been obtained for higher molecular weight (i.e., neddylated) forms of δ subunit in postsynaptic nAChR isolated from muscle or agrin-treated muscle cell cultures or for neddylated proteins being localized at a high density at the NMJ. Consequently, it will be important to determine whether neddylation of the δ subunit regulates other processes such as trafficking and turnover of the nAChR, and if other proteins are also neddylated by rapsyn that are important for clustering. Interestingly, the major neddylation site on δ lies between a highly conserved agrin-induced tyrosine phosphorylation site (−4 amino-acids) and two CMS mis-sense mutations which impair expression and/or clustering of receptor (+2 and +8 amino-acids). This identifies this highly conserved region of the δ subunit loop as a possible component of a rapsyn binding site or a region that modulates rapsyn binding to adjacent subunit loops.

In summary, several independent lines of evidence have converged to show that multiple rapsyn molecules bind the nAChR subunit cytoplasmic loops to localize the receptor in the postsynaptic membrane. The stoichiometry of rapsyn/nAChR complexes is variable and regulated by agrin signaling, allowing for fine control over AChR clustering, anchoring, and stability. Several important questions remain, however, including which subunits or subunit interfaces are bound by rapsyn, and whether rapsyn binding to different sites nucleates specific rapsyn–scaffolding protein linkages with distinct functions.

## 4. Postsynaptic Localization of Neuronal nAChRs

The neuronal nAChR occurs in multiple subtypes with different subunit compositions that confer distinct functional properties and possibly different subcellular localizations. In the vertebrate CNS, neuronal nAChRs have diverse and mainly neuromodulatory functions, and are often localized on presynaptic nerve terminals where they modulate the transmitter release. In the PNS, however, neuronal nAChRs mediate fast synaptic transmission at inter-neuronal synapses and are essential for autonomic function. These fast cholinergic synapses are mostly located in autonomic ganglia, where visceral motoneurons (preganglionic neurons) synapse onto sympathetic or parasympathetic postganglionic neurons. At these synapses, the neuronal nAChR is aggregated at a high density in the postsynaptic membrane and is composed predominantly of α3, α5, β2, and β4 subunits [97]. Notably, mice with a knockout of the α3 subunit or double knockout of the β2 and β4 subunits have severely reduced ganglionic transmission and multiorgan autonomic dysfunction, leading to significant postnatal mortality [98,99,100]. Homo-oligomers composed of α7 subunits also contribute to transmission at some peripheral synapses [101].

Although ganglionic synapses are functionally similar to neuromuscular synapses, surprisingly few parallels have been described regarding the signals that regulate their development or the scaffolding proteins that localize the nAChR at each synapse. Agrin’s role is partially conserved as it helps direct neuronal synapse formation in sympathetic ganglia and adrenal medulla. Most notably, synaptic differentiation and transmission are impaired in superior cervical ganglia of agrin null mice [102,103], and agrin rapidly increases the strength of nicotinic transmission at developing sympathetic synapses on adrenal chromaffin cells by recruiting additional nAChRs to postsynaptic sites [104]. Synapses are not eliminated in agrin null ganglia as they are at the NMJ, however, indicating that additional synaptogenic factors contribute to the formation of cholinergic inter-neuronal synapses [105].

### Determinants for Neuronal nAChR Localization

**Figure 3:**
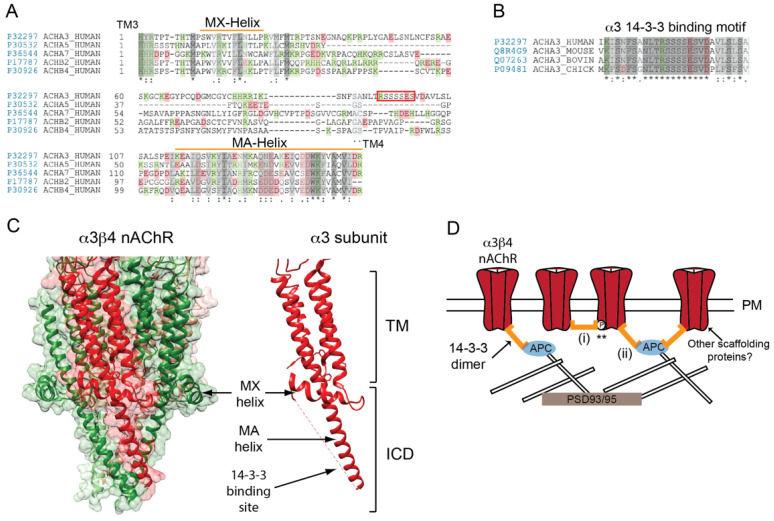
Determinants of neuronal nAChR postsynaptic localization. (**A**) A sequence alignment of the neuronal (ganglionic-type) nAChR subunit large cytoplasmic loops between transmembrane (TM) domains 3 and 4. The subunit loops share a moderate homology in their proximal and distal portions, but differ considerably in the central region. (**B**) The putative 14-3-3 binding motif in the mid-portion of α3 subunit loop is highly conserved across species (identical residues are denoted by * and conserved and semi-conserved substitutions by : and . respectively). (**C**) A high resolution structure of the neuronal α3β4 AChR (PDB: 6PV7) showing the transmembrane and intracellular domains. The large cytoplasmic loop of neuronal subunits has a similar structure to that of muscle nAChR subunits and contain analogous MX- and MA-α-helices. (**D**) A speculative model for neuronal nAChR localization at ganglionic synapses. Binding of the 14-3-3 adaptor protein to the α3 subunit loop clusters the receptor in the postsynaptic membrane via 14-3-3 dimerization (i) and/or binding to APC and its associated cytoskeletal proteins (ii). Potentially, two 14-3-3 molecules could interact with the nAChR (one to each α3 subunit) and binding could be regulated by phosphorylation of the α3 loop motif (**). Other scaffolding proteins could also contribute to receptor localization.

Downstream of these synapse-organizing factors, the molecular basis for neuronal nAChR localization at ganglionic synapses is poorly defined. This question was first addressed using chimeric nAChRs to identify the receptor subunits that mediated synaptic localization. In an elegant study, Michele Jacob’s group showed that chimeric α7 nAChRs that contained the α3 but not α5 or β4 large cytoplasmic loops were retargeted from peri-synaptic regions to the postsynaptic membrane [106,107]. This demonstrates that the α3 cytoplasmic loop is sufficient for synaptic localization (Figure 3A), although as for the muscle nAChR, it has proved challenging to define the motifs involved and to confirm that they are required for receptor localization. One intriguing possibility is a conserved consensus motif in α3 loop for binding of the 14-3-3 adaptor protein (Figure 3B,C). Indeed, 14-3-3 partially colocalizes with the nAChR at synapses in chick ciliary ganglia and associates biochemically with the receptor both in vivo and in vitro [108]. The 14-3-3 protein also interacts with adenomatous polyposis coli (APC), which organizes a postsynaptic scaffold that includes several microtubule-associated proteins and is important for nAChR surface expression [108,109]. Thus, 14-3-3 could link α3-nAChR to APC and thereby localize the nAChR in the postsynaptic membrane (Figure 3C). As 14-3-3 binding is often phosphorylation-dependent, phosphorylation of conserved serine residues in the motif could regulate binding of two 14-3-3 molecules to the nAChR (i.e., one to each of the α3 subunits in the pentamer). Moreover, as 14-3-3 proteins commonly form dimers, they could cross-link and cluster neuronal nAChRs in a manner analogous to rapsyn-induced clustering of muscle nAChRs (Figure 3D). While an attractive possibility, it remains to be shown that the 14-3-3 binding motif in α3 loop is either necessary and/or sufficient for receptor localization.

In addition, based on the muscle nAChR model, it is possible that additional interactions could contribute to postsynaptic localization of neuronal nAChRs. For instance, other scaffolding proteins could bind the MA helix of one or more receptor subunits, in a manner analogous to rapsyn binding to the muscle nAChR. Interestingly, rapsyn can cluster neuronal nAChRs in heterologous cells but it is not localized at ganglionic synapses and nAChR remains aggregated at these synapses in rapsyn null mice [83,84]. Alternative scaffolding proteins include PSD93 and 95, which are both localized at ganglionic synapses, however they do not bind neuronal nAChRs directly and are dispensable for nAChR localization [110,111]. Similarly, APC does not bind neuronal α3β4 nAChRs directly [109], even though it binds muscle nAChRs via the β1 subunit MA-helix [87]. Thus, APC and PSD93/95 help organize the postsynaptic scaffold rather than directly anchoring nAChRs at the synapse.

In summary: Many questions remain on how neuronal nAChRs are localized at cholinergic inter-neuronal synapses in the PNS. Most notably, is the α3 subunit 14-3-3 binding motif required for nAChR localization at synapses, and do additional molecular determinants also contribute to receptor localization? Is nAChR localization regulated by phosphorylation and does this control the stoichiometry of 14-3-3/nAChR interactions? In addition, what are the key scaffolding protein(s) that anchor 14-3-3/nAChR complexes at PNS synapses? These questions become even more complex when considering nAChR localization in the CNS, as multiple nAChR subtypes with differing subunit compositions are located at diverse subcellular locations on neurons. It seems likely that an array of subunit-specific molecular mechanisms are employed to regulate neuronal nAChR localization in vertebrate neurons, and this would presumably involve distinct binding proteins. Unraveling these diverse mechanisms remain an important challenge which is critical to understanding the nAChR function in both the PNS and CNS.

## Abbreviations

APC	adenomatous polyposis coli
CNS	central nervous system
CMS	congenital myasthenic syndrome
ICD	intracellular domain
nAChR	nicotinic acetylcholine receptor
NMJ	neuromuscular junction
PNS	peripheral nervous system
PM	plasma membrane
TM	transmembrane
